# Interface Modulation of Core-Shell Structured BaTiO_3_@polyaniline for Novel Dielectric Materials from Its Nanocomposite with Polyarylene Ether Nitrile

**DOI:** 10.3390/polym10121378

**Published:** 2018-12-12

**Authors:** Yong You, Yajie Wang, Ling Tu, Lifen Tong, Renbo Wei, Xiaobo Liu

**Affiliations:** Research Branch of Advanced Functional Materials, School of Materials and Energy, University of Electronic Science and Technology of China, Chengdu 611731, China; yourkeaib@163.com (Y.Y.); m18380127314@163.com (Y.W.); tutu576695@163.com (L.T.); tonglifen@163.com (L.T.)

**Keywords:** nanoparticles, nanocomposites, in-situ polymerization, dielectric properties

## Abstract

The core-shell structured polyaniline-*functionalized*-BaTiO_3_ (BT@PANI) nanoparticles with controllable shell layer thicknesses are developed via in-situ aniline polymerization technology and characterized in detail. The results prove that the PANI shell layer with the adjustable and controllable thicknesses of 3–10 nm are completely stabilized on the surface of the BaTiO_3_ core. In addition, the BT@PANI nanoparticles are regarded as the hybrid nanofillers to prepare PEN/BT@PANI nanocomposite films with a PEN matrix. The research results indicate that the surface functionalized nanoparticles facilitate the compatibility and dispersibility of them in the PEN matrix, which improves the properties of the PEN/BT@PANI nanocomposites. Specifically, the PEN/BT@PANI nanocomposites exhibit thermal stability, excellent permittivity-frequency, and dielectric properties-temperature stability. Most importantly, the energy density of nanocomposites is maintained at over 70% at 180 °C compared with that at 25 °C. All these results reveal that a new way to prepare the high-performance PEN-based nanocomposites is established to fabricate an energy storage component in a high temperature environment.

## 1. Introduction

Nowadays, with the constantly increasing demand of microelectronic devices, high-temperature-resistant, integrated, and flexible electronic materials have attracted wide interest from researchers for their application in harsh environments [1,2,3]. However, so far, it is difficult for a single component material to completely fulfil the above requirements. Therefore, developing multi-component composites is an important path to overcome these defects. Recently, polymer-based nanocomposites have been proverbially used as film capacitors with a high energy density in the electronics industry fields by the scientific research community in virtue of their excellent properties, such as their light weight, flexibility, high permittivity, and outstanding thermal stability [4,5]. This is mainly attributed to the fact that the polymer-based nanocomposites achieve a high thermal stability and flexibility from the polymer matrix and high dielectric properties from the nanofillers [6,7]. 

At present, in order to obtain high permittivity composites, some conductive particles, including carbon nanotube (CNT) [8,9], grapheme [10], metal particles [11], etc., have been utilized to prepare polymer-based composites according to previous reports. However, these conductive particles can effectively enhance the permittivity of the polymer matrix and inevitably increase their dielectric loss, which is extremely harmful to energy storage components. Consequently, the ferroelectric ceramic particles are a new choice to improve the dielectric properties of the polymer matrix in view of their unique ferroelectricity, high permittivity, and ultralow dielectric loss [12,13,14]. Barium titanate (BaTiO_3_), a typical ferroelectric ceramic, has been extensively used as nanofillers to heighten the dielectric properties of the polymer matrix [15,16]. However, BaTiO_3_ nanoparticles are always vulnerable to local agglomeration due to their high surface energy, which causes poor compatibility with the matrix, resulting in an awful comprehensive performance of the composites [17,18]. To overcome this drawback, modifying the surface of the nanoparticles can greatly promote their compatibility with the polymer matrix [19,20]. Although surface decoration is an effective pathway, the choices of methods in modification for achieving the aforementioned performance are still a challenge [21]. On one hand, the energy of nanoparticles should be reduced effectively in order to promote their uniform dispersion in the matrix and against local agglomeration through surface functionalization. The common surface modification techniques include physical coating, surface chemical grafting, and in situ growth, etc. On the other hand, the regulation of micro dimensions should on the basis of maintaining the properties of nanoparticles while improving the compatibility. 

As a high-performance thermoplastic, Polyarylene ether nitrile (PEN) is regarded as a research hotspot in view of its highlighted formability, flexibility, thermal stability, chemical resistance, and mechanical properties [22,23,24]. The core-shell structured BT@PANI nanofillers with controllable shell layer thickness are developed via in-situ aniline polymerization technology in this work, and BT@PANI nanoparticles are then regarded as the hybrid nanofillers to prepare PEN/BT@PANI nanocomposite films. The corresponding thermal, mechanical, and dielectric properties, as well as energy storage characteristics, are studied systematically.

## 2. Materials and Methods

### 2.1. Materials

BaTiO_3_ (<100 nm) was supplied by TPL Co., Ltd. (Texas, USA). Aniline (C_6_H_7_N), ammonium persulfate ((NH_4_)_2_S_2_O_8_), 2,6-dichlorobenzonitrile (DCBN), biphenol (BP), potassium carbonate (K_2_CO_3_), acetone, alcohol, toluene, and hydrochloric acid were bought from Chengdu KeLong chemicals (Chengdu, China). *N*-Methyl-2-pyrrolodone (NMP) was supplied by Chengdu Changzheng chemicals (Chengdu, China).

### 2.2. Synthesis of PEN

Polyarylene ether nitrile (PEN) was synthesized by 2,6-dichlorobenzonitrile and biphenol through nucleophilic aromatic substitution polymerization, according to the previously reported method [24]. The corresponding synthesis route and process are shown in Figure S1.

### 2.3. In-Situ Preparation of Surface Functionalized BaTiO_3_ Nanoparticles

**Figure 1:**
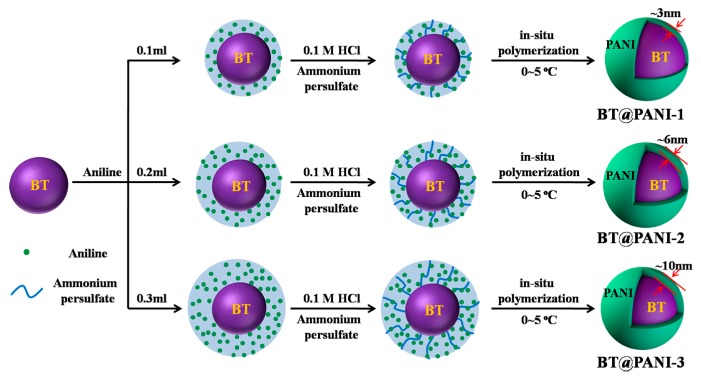
Schematic diagram of core-shell structured BT@PANI nanoparticles.

The BaTiO_3_@polyaniline (BT@PANI) nanoparticles were prepared through in-situ polymerization technology [25]. The corresponding preparation diagram is shown in Figure 1. In this work, the molar ratio of aniline to ammonium persulfate was 1:1.2, and the amounts of aniline of 0.1, 0.2, and 0.3 mL are named BT@PANI-1, BT@PANI-2, and BT@PANI-3, respectively. A typical prepared method was employed, as follows: BaTiO_3_ (<100 nm, 1.0 g) was firstly dispersed in 100 mL deionized water with continuous ultrasonicating for 1 h in an ice-water environment. Then, the aniline pre-cooled in the ice-water bath was dissolved in 50 mL 0.1 M HCl, and the aniline/HCl solution was tardily added into the system. After that, the pre-cooled ammonium persulfate/deionized water solution was added into the mixture for oxidative polymerization for 18 h at 0~5 °C to obtain the corresponding BT@PANI nanoparticles.

### 2.4. Preparation of PEN/BT@PANI Nanocomposite Films

The PEN-based multi-component nanocomposite films with 0, 5, 10, 20, and 40 wt% BT@PANI nanoparticles were fabricated through a solution casting method based on the reported method [24] (PEN/BT@PANI 0, PEN/BT@PANI 5, PEN/BT@PANI 10, PEN/BT@PANI 20, and PEN/BT@PANI 40, respectively). In addition, the 40 wt% pure BT was also filled in the PEN matrix (PEN/BT 40) for comparison.

### 2.5. Characterization

The composition and chemical structure of nanoparticles were characterized by XPS (ESCA 2000, VG Microtech, Sussex, UK) and FTIR (8400S, Shimadzu, Tokyo, Japan). The component was analyzed through XRD (RINT2400, Rigaku, Tokyo, Japan) and UV-vis (TU1800, Beijing Purkinje General Instrument, Beijing, China). The microstructures and EDS elemental mappings of the composites were tested by SEM (6490LV, JSM, Tokyo, Japan). Thermal properties of samples were carried out in N_2_ atmosphere by DSC (Q100, TA Instruments, New Castle, DE, USA) and TGA (Q50, TA Instruments, New Castle, DE, USA). Mechanical properties of the composites were performed using the universal testing machine (CMT6104, SANS, Shenzhen, China). Electric breakdown strengths of composites were measured through a Dielectric Withstand Voltage Tester (ZJC-50KV, Zhonghang Shidai, Beijing, China). Dielectric performances of composites were investigated on a precision LCR meter (TH 2819A, Tong hui, Dongguan, China).

## 3. Results and Discussion

BT@PANI nanoparticles with different shell layer thickness were prepared via the aniline polymerization method, and detailed characterizations of BT@PANI nanoparticles were studied in this work. The TGA and UV-vis results show that the PANI with different contents has been successfully prepared on the surface of BT nanoparticles, and the shell layer weight losses of samples are 4.61%, 8.70%, and 15.18%, respectively (Figure S1). Additionally, in view of the different contents of PANI at the BT@PANI nanoparticles reflected by the thickness of the shell layers, it can be directly observed through the TEM images. As shown in Figure S1, the uniform thickness of the polyaniline layer on the surface of BT nanoparticles increases from 3 nm to 10 nm with the increase of aniline content. In general, the morphology variation and size of nanoparticles have a great influence on the properties (especially the electrical properties) of the polymer-based composites [26]. Therefore, 20 wt% of nanoparticles with different shell thickness are used as fillers to fill in the PEN matrix, and their electrical properties are studied in this system. As shown in Table S1, the electrical performance results show that the PEN/BT@PANI-2 nanocomposites exhibit the best energy storage properties among the samples. Therefore, BT@PANI-2 is used as the object for further study, and the effect of its content (0, 5, 10, 20, 40 wt%) in the PEN matrix on the performances of composite films is amply researched in the rest of the work.

### 3.1. Characterization of BT@PANI Nanoparticles

**Figure 2:**
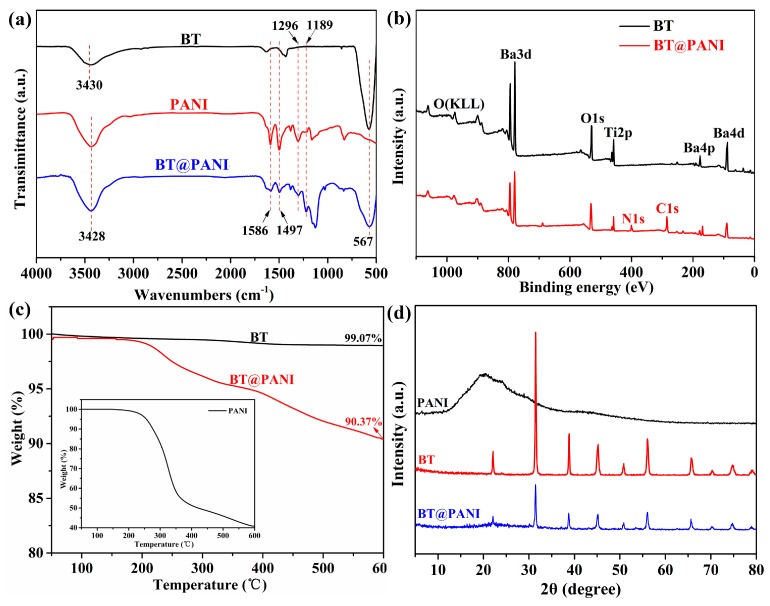
The characterization of nanoparticles: (**a**) FTIR spectrum; (**b**) XPS spectrum; (**c**) TGA curves; (**d**) XRD patterns.

The chemical structure and composition of nanoparticles were characterized by FTIR and XPS. Figure 2a shows the FTIR spectra of nanoparticles. It is obvious that the band at 567 cm^−1^ belongs to the vibration of Ti–O [27]. Besides, two additional absorption peaks at 1497 and 1586 cm^−1^, belonging to the skeleton vibration of benzene rings, can be found in the curves of PANI and BT@PANI nanoparticles. What is more, the absorption bands at 1189 and 3428 cm^−1^ are found in curve of BT@PANI by comparing them with BT, which are the absorption peaks of Ar–N and N–H vibrations, respectively. It is indicated that polyaniline exists in the BT@PANI nanoparticles [21]. In addition, Figure 2b shows the XPS spectrums of the obtained BT and BT@PANI nanoparticles. In comparison with pure BT, it is clearly found that the two new peaks at 286 eV and 402 eV appeared at the spectrum of BT@PANI nanoparticles, corresponding to C1s and N1s from polyaniline. Therefore, both FTIR and XPS measurements proved that the PANI is successfully grown on the surface of the BT nanoparticle by the in-situ polymerization method.

After the successful fabrication of core-shell structured BT@PANI nanoparticles, the content of polyaniline from the shell structure in the BT@PANI nanoparticles was determined by the TGA test. As shown in Figure 2c, it is clear that the TGA curves of BT@PANI have an obvious weight decrement from 250 °C, which is seen to represent the decomposition of polyaniline by comparing it with the curve of the pure polyaniline (inset). Besides, the X-ray diffraction (XRD) is further used to confirm the component of BT@PANI nanoparticles (Figure 2d). It is obvious that the characteristic absorption peaks of BT are reserved in BT@PANI nanoparticles. In addition, an extra weak steamed bread peak at around 22°, belonging to the characteristic absorption peak of PANI [28], appears in the prepared core-shell-structured nanoparticles by comparing it with the curves of pristine BT. These results confirm that the PANI with different contents has been successfully prepared on the surface of BT nanoparticles by in-situ polymerization technology.

**Figure 3:**
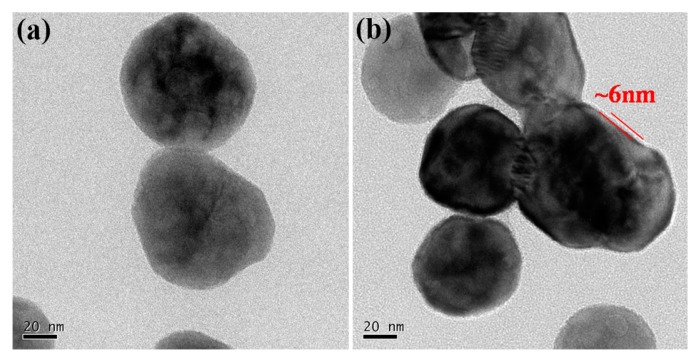
TEM images of nanoparticles: (**a**) BT and (**b**) BT@PANI.

The TEM micro-images of the pristine BT and core-shell structured BT@PANI nanoparticles are shown in Figure 3, which is also an approach to further intuitively prove the surface functionalization. As shown in Figure 3a, it is not difficult to see that the pristine BT nanoparticles present a bare surface and a smooth edge. Nevertheless, the polymer layer can be clearly observed on the surface of prepared core-shell structured BT@PANI nanoparticles, which are shown in Figure 3b. Specifically, the uniform thickness of the polyaniline layer is about 6 nm. Therefore, the TEM micro-images further prove that surface functionalized BT nanoparticles are developed by in-situ polymerization technology.

### 3.2. Morphology of the Nanocomposite Films

**Figure 4:**
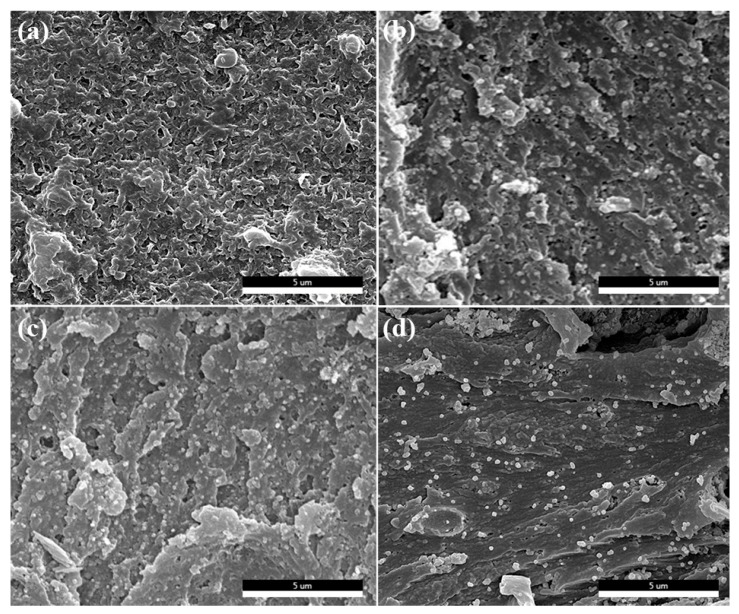
Cross-sectional SEM images of nanocomposite films: (**a**) pure PEN; (**b**) PEN/BT@PANI 5; (**c**) PEN/BT@PANI 40; (**d**) PEN/BT 40.

It is well-known that the interfacial microstructure between the nanofillers and matrix is an important diathesis that affects the performances of polymer-based nanocomposites, like thermal, mechanical, and dielectric properties, etc. The dispersability and miscibility of nanofillers in the polymer matrix can be observed intuitively by SEM micro-images [27]. The cross-sectional microstructures of nanocomposites are displayed in Figure 4. It is obvious that PEN/BT@PANI 0 (Figure 4a) presents a typical ductile fracture, indicating that the PEN matrix is a kind of thermoplastic engineering material with a high toughness. Besides, PEN/BT@PANI 5 shows that the inorganic nanofillers are uniformly distributed in the polymer matrix, and no obvious phase separation can be discovered between the organic and the inorganic phase (Figure 4b). In addition, when the content of nanoparticles increases to 40 wt%, there is still good compatibility between the BT@PANI and PEN matrix, even though a few nanoparticles show a slight tendency of local agglomeration (Figure 4c). This is mainly attributed to the following: firstly, the benzene ring structure of PANI is similar to the molecular structure of the PEN matrix, so they have good compatibility with each other according to the similar compatibility principle; secondly, the macromolecular chains of PANI and the PEN matrix can further improve their compatibility through physical entanglement; thirdly, the PANI layer can effectively reduce the surface energy of BT nanoparticles, resulting in reducing its agglomeration in the matrix [29,30]. Contrarily, it can be obviously seen that the pristine BT has a large amount of agglomeration and nudity in the PEN matrix from the cross-section morphology of PEN/BT 40 (Figure 4d), revealing poor compatibility and dispersibility between the BT and PEN. This is because the high surface energy of pristine BT nanoparticles enables it to easily agglomerate in the PEN matrix [29]. Therefore, in this system, the surface functionalized PANI layer can be regarded as a “molecular bridge” between inorganic and organic phases, which can effectively promote the dispersibility and compatibility of core-shell structured inorganic nanofillers in polymer substrates [31].

**Figure 5:**
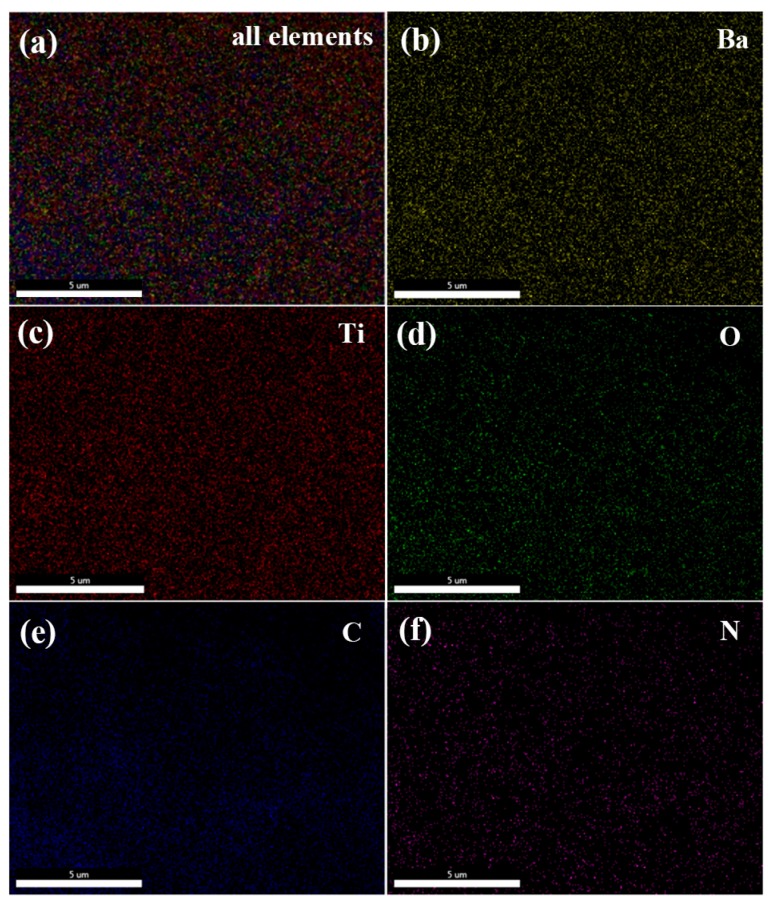
SEM EDS mapping images of (**a**) all elements, (**b**) Ba, (**c**) Ti, (**d**) O, (**e**) C, and (**f**) N.

In addition, to further verify the dispersibility of BT@PANI nanoparticles in the PEN matrix, the cross-sectional SEM images of PEN/BT@PANI 40 (Figure 4c) were further characterized by energy dispersive spectroscopy, and are shown in Figure 5. As mentioned, the color distribution points of different elements are homogeneously distributed in the all elements mapping (Figure 5a), indicating that the inorganic nanofillers are uniformly dispersed in the matrix. Besides, the barium (Ba, Figure 5b), titanium (Ti, Figure 5c), and oxygen (O, Figure 5d) elements, belonging to BT, are evenly present in the PEN matrix, even with a high nanoparticles content, which are all observed in the SEM EDS mapping. Similarly, carbon (C, Figure 5e) and nitrogen (N, Figure 5f) elements from PANI and PEN are also found in the images. This further confirmed the excellent dispersibility of inorganic nanoparticles in the polymer matrix, indicating that surface functionalization of nanoparticles is a valid way to enhance the compatibility between BT@PANI nanoparticles and the PEN matrix.

### 3.3. Thermal Properties of the Nanocomposite Films

**Figure 6:**
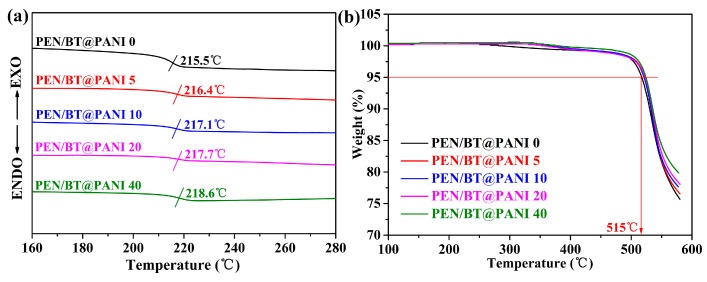
Thermal properties of the nanocomposite films: (**a**) DSC curves; (**b**) TGA curves.

The thermal properties of polymer-based composite films are an important index to evaluate the actual operating temperature of the films. Normally, the thermal transition temperatures of polymer-based composites are obtained through the DSC test. Figure 6a processes the DSC curves of PEN/BT@PANI nanocomposites. It is obvious that all samples present a high glass transition temperature (*T*_g_), with all exceeding 215 °C. As to details, the *T*_g_s of PEN/BT@PANI nanocomposites increase gradually from 215.5 to 218.6 °C as the BT@PANI nanoparticles content increases from 0 to 40 wt%. This is mainly attributed to two factors: on the one hand, molecular entanglement occurs between macromolecular chains from PANI on the surface of BT and macromolecular chains of the PEN matrix, limiting the movement of the main chains of the nanocomposites; on the other hand, the rigid nanoparticles are uniformly dispersed in the polymer matrix, which will form a strong bonding effect between inorganic nanoparticles and the polymer matrix, resulting in the restriction of molecular chains movement. Besides, the movement of the molecular chain is further blocked with the increasing of BT@PANI nanoparticles content, which leads to the increase of the *T*_g_. [24,29]. In addition, the thermal stability of PEN/BT@PANI nanocomposites is measured through the TGA instruments, and the corresponding curves are shown in Figure 6b. It is clear that the 5% initial decomposition temperatures (*T*_5%_) of nanocomposite films are over 510 °C in the flowing nitrogen, showing the excellent thermal resistance. This is due to the outstanding thermal resistance of PEN. Therefore, these results approve that the PEN/BT@PANI nanocomposites have a huge potential to be used in the high temperature environment.

### 3.4. Mechanical Properties of the Nanocomposite Films

**Figure 7:**
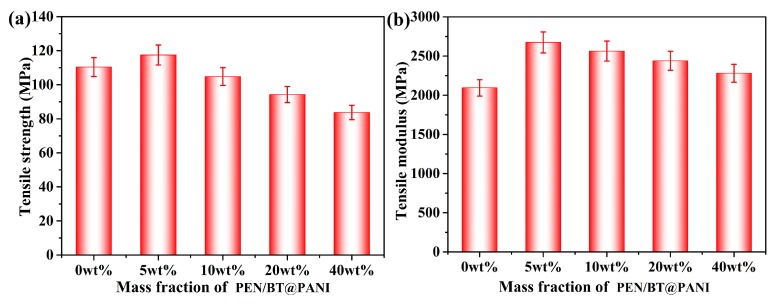
Mechanical properties of the nanocomposite films: (**a**) tensile strength; (**b**) tensile modulus.

PEN/BT@PANI nanocomposite films, as a high-performance polymer-based composite material, present excellent mechanical properties. It is clear that the tensile strength of PEN/BT@PANI nanocomposite films firstly displays a slight increase and then shows a gradual downward trend as the nanoparticles increase continuously (Figure 7a). Concretely, the tensile strength of PEN/BT@PANI 5 is 117.5 MPa, which is 6.4% higher than that of pure PEN film (110.4 MPa). This is because the surface functionalized method can improve the compatibility between the BT@PANI and PEN matrix effectively and the nanofillers can be dispersed uniformly in the PEN matrix at a low filling constant. In addition, it is not difficult to see that the tensile strengths of PEN/BT@PANI nanocomposite films in this work are all over 83 MPa, which is much higher than the commercial dielectrics, such as PP and PVDF. Moreover, the tensile modulus of PEN/BT@PANI 40 (2280.2 MPa) is slightly higher than that of pure PEN (2094.0 MPa), which is shown in Figure 7b. Therefore, these results prove that the PEN/BT@PANI nanocomposite films still maintain relatively good mechanical properties to meet the practical application.

### 3.5. Dielectric Properties of the Nanocomposite Films

**Figure 8:**
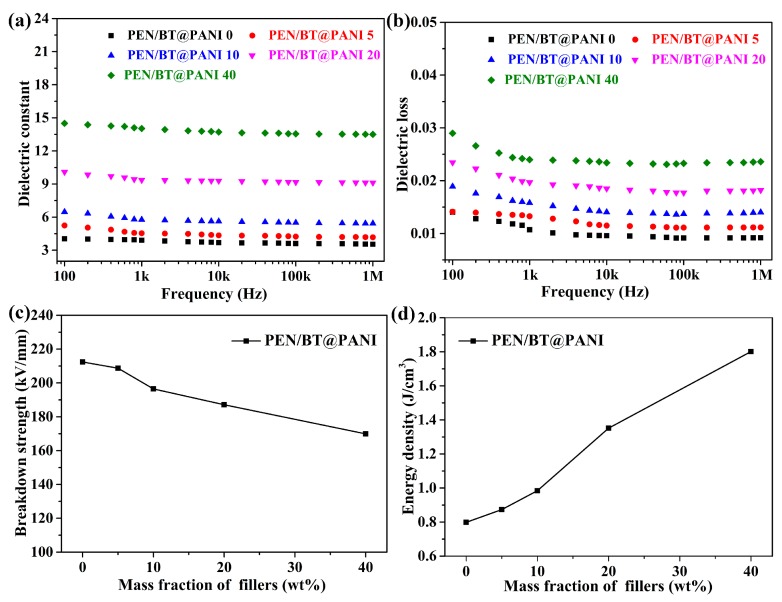
The (**a**) dielectric constant, (**b**) dielectric loss, (**c**) breakdown strength, and (**d**) energy density at 1 kHz of the nanocomposite films.

In order to study the dielectric behavior of PEN-based nanocomposites with different contents of nanofillers, a series of different contents of BT@PANI nanoparticles were used to fill in the PEN matrix. The dielectric properties-frequency-dependence of PEN-based nanocomposite films were measured with a varying frequency from 100 Hz to 1 MHz. Figure 8a processes the permittivity of the nanocomposites with different contents of BT@PANI nanoparticles. It is clear that the permittivity of all nanocomposite films shows a slight trend of depression as the frequency increases, which is caused by a polarization relaxation process [29]. However, the fluctuation range of the PEN/BT@PANI nanocomposite films on dielectric constant is within 7% from 100 Hz to 1 MHz. Even when the BT@PANI nanoparticles content is 40 wt% (PEN/BT@PANI 40), it is only 6.5%. This result reveals that the PEN/BT@PANI nanocomposites present a good dielectric constant-frequency stability, which is due to the better compatibility between the core-shell structured nanoparticles and PEN matrix [24]. Besides, it is easy to find that the permittivity of nanocomposites increases gradually as the BT@PANI content increases. The dielectric constant of PEN/BT@PANI 40 is 14.0, which is 3.59 times as much as that of the pure PEN matrix (3.9) at 1 kHz. The variations of permittivity with different contents of nanocomposites are shown in Figure S3a. This is due to the following: firstly, the homogeneous inorganic nanoparticles in nanocomposites can be considered as a micro-capacitor network, and its number increases gradually as the content of nanoparticles increases [28]; secondly, the permittivity of inorganic nanoparticles is much higher than that of the PEN matrix, which leads to a large amount of free charge gathering at the interface between the fillers and PEN matrix, resulting in a high permittivity of PEN/BT@PANI nanocomposites [32]. Generally, the dielectric loss is the essential diathesis that affects the energy dissipation in practical applications, and the high dielectric loss is detrimental to the electron device. As shown in Figure 8b, the dielectric loss of nanocomposites shows a similar trend to the dielectric constant, presenting a slight increase as the BT@PANI content increases. However, it is still lower than 0.025 at 1 kHz, even though the BT@PANI nanoparticle content is 40 wt% (PEN/BT@PANI 40), which is also at a relatively low value to satisfy the requirements of practical applications (Figure S3b) [33,34]. This is attributed to core-shell structured nanoparticles, which can promote their compatibility with the matrix and their dispersibility in the matrix. What is more, the energy density of nanocomposites is another important parameter for use in electronic component fields. The energy density (*U*) of linear dielectrics is calculated by Equation (1):

(1) $$U=\frac{1}{2}\varepsilon_{0}\varepsilon_{r}{E_{b}}^{2}$$

where *ε*_0_ is the vacuum perpitivity, and *ε*_r_ and *E*_b_ are the realistic permittivity and the breakdown strength of nanocomposites, respectively. It can be seen from Equation (1) that the energy density is determined by both the permittivity and the breakdown strength. Therefore, the breakdown strengths of PEN-based nanocomposites with different nanoparticles contents are measured in detail. As shown in Figure 8c, the breakdown strength of PEN/BT@PANI nanocomposite films has a gradual downward trend as the BT@PANI nanoparticles content increases, which decreases from 212.4 to 169.8 kV/mm at 25 °C. This is caused by the increase of nanoparticles content, which causes free charge gathering at the interface between the fillers and PEN matrix, resulting in a gradual decrease in breakdown strength. Moreover, the results of energy density, calculated by Equation (1), show that the energy density of PEN/BT@PANI nanocomposites increases gradually from 0.8 to 1.8 J/cm^3^ as the content of BT@PANI nanoparticles increases from 0 to 40 wt% (Figure 8d). All these results indicate that the PEN/BT@PANI nanocomposites exhibit huge potential for applications in the film capacitors field.

**Figure 9:**
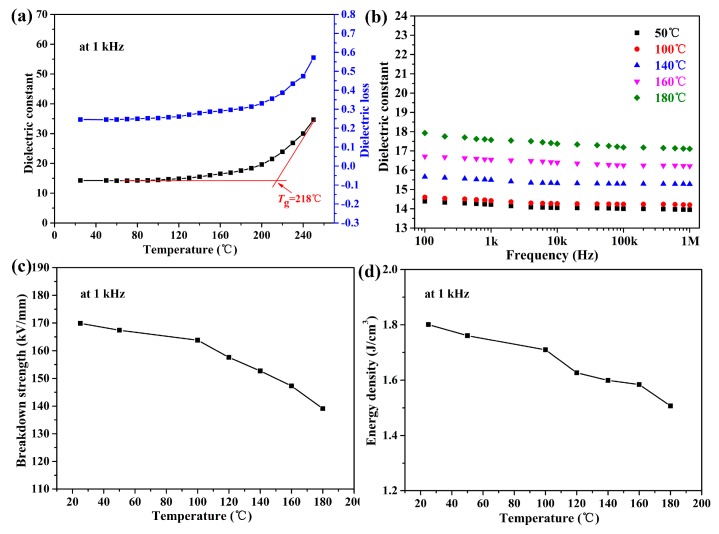
The energy storage characteristics of the PEN/BT@PANI 40 composite films: (**a**,**c**,**d**) are the dielectric constant, breakdown strength, and energy density at 1 kHz, respectively; (b) the dielectric constant at different temperatures.

Furthermore, the dielectric properties-temperature dependence of PEN/BT@PANI 40 was deeply studied at 1 kHz from 50 to 250 °C. Wei et al. has proved the long-term use of PANI/PEN composites at 180 °C by testing their dielectric properties [35]. In this study, the dielectric permittivity is higher and loss is more stable before the *T*_g_ of the nanocomposites when compared with PANI/PEN, as shown in Figure 9a. This result is caused by the macromolecular motion of the PEN matrix, which is suppressed before the *T*_g_ and then moves violently after *T*_g_, resulting in the increasing polarization inside the system [36]. Besides, the permittivity of PEN/BT@PANI 40 is fairly stable, with a varying frequency from 100 Hz to 1 MHz at different temperatures, which are shown in Figure 9b. It reveals that the PEN/BT@PANI 40 exhibits an excellent permittivity-frequency stability at high temperature. In addition, Figure 9c shows the breakdown strength of PEN/BT@PANI 40 with a varying temperature. It demonstrates that the breakdown strength of PEN/BT@PANI 40 decreases from 169.8 to 139.1 kV/mm as the temperature increases from 25 to 180 °C. More importantly, the energy density of PEN/BT@PANI 40 is still 1.5 J/cm^3^, even at 180 °C (Figure 9d). The results indicate that the PEN/BT@PANI nanocomposite films are greatly significant for use as the energy storage component in high temperature environments.

## 4. Conclusions

In summary, the core-shell structured BT@PANI nanoparticles with controllable shell layer thicknesses are developed via in-situ aniline polymerization technology in this work, and BT@PANI nanoparticles are then regarded as hybrid nanofillers to prepare PEN/BT@PANI nanocomposite films. The results prove that the PANI layer, modified on the surface of BaTiO_3_ nanoparticles, can be adjusted and controlled through in-situ polymerization technology, and the BT@PANI nanoparticles with a 6 nm polymer layer exhibit the best performance in this system. In addition, the surface functionalized nanoparticles realize their good compatibility and dispersibility in the PEN matrix, which can improve the thermal and dielectric properties of the PEN/BT@PANI nanocomposites. The research results reveal that the PEN/BT@PANI nanocomposites present excellent thermal stability (*T*_g_ > 215 °C and *T*_5%_ > 510 °C). In addition, the PEN/BT@PANI nanocomposites also exhibit a relatively high permittivity (~14 at 1 kHz), and excellent dielectric properties-temperature stability (25~180 °C). Most importantly, the energy density of nanocomposites is still able to be maintained at over 80%, even at 180 °C, by comparing it with that at 25 °C. All these results prove that the PEN/BT@PANI nanocomposite films are greatly significant for use as an energy storage component in high temperature environments.

## Supplementary Materials

The following are available online at http://www.mdpi.com/2073-4360/10/12/1378/s1, Figure S1: Synthetic route to the PEN, Figure S2: The characterization of nanoparticles: (a) TGA curves (I: BT, II: BT@PANI-1, III: BT@PANI-2, IV: BT@PANI-3); (b) UV-vis spectra; TEM image of (c) BT; (d) BT@PANI-1; (e) BT@PANI-2; (f) BT@PANI-3, Figure S3: The dielectric constant (a) and dielectric loss (b) at 1 kHz of the PEN based nanocomposite films, Table S1: The dielectric constant, dielectric loss, breakdown strength and energy density of the PEN/BT@PANI composite films at 1 kHz (20 wt%).
