# Supplementary material for: Interface Modulation of Core-Shell Structured BaTiO3@polyaniline for Novel Dielectric Materials from Its Nanocomposite with Polyarylene Ether Nitrile
Source: Polymers (Basel). 2018 Dec 12;10(12):1378. doi: 10.3390/polym10121378 (PMC6401899; doi:10.3390/polym10121378)
Supplement: Supplementary file 1 [file polymers-10-01378-s001.pdf]

Supplementary Information

Interface modulation of core-shell structured $BaTiO_3$@polyaniline for novel dielectric materials from its nanocomposite with polyarylene ether nitrile

Yong You, Yajie Wang, Ling Tu, Lifen Tong, Renbo Wei* and Xiaobo Liu*

Research Branch of Advanced Functional Materials, School of Materials and Energy, University of Electronic Science and Technology of China, Chengdu 610054, China

Corresponding author. Tel: +86-28-83207326; Fax: +86-28-83207326; E-mail address: weirb10@uestc.edu.cn
Corresponding author. Tel: +86-28-83207326; Fax: +86-28-83207326; E-mail address: liuxb@uestc.edu.cn

Synthesis of PEN: NMP (150 mL), toluene (50 mL), BP (37.2 g), DCBN (34.4 g), K_2CO_3 (34.7 g) were sequentially added into a 500 mL three-necked round-bottom flask equipped with a Dean-Stark trap and a mechanical stirrer. Then the reaction was maintained at 145 °C for 3 h for dehydration. After the water-toluene azeotrope distilled off, the reaction mixture was heated to 160, 170, and 180 °C for 1 h, respectively. Then, the product was precipitated by precipitated into water, and the precipitate was smashed by a pulverizer. After that, the product was poured into 1000 mL of diluted HCl solution in order to remove the excess K_2CO_3, and then rinsed by acetone and alcohol till the solvent and monomers were washed out completely. Finally, the product was dried at 100 °C in a vacuum oven for 12 h.

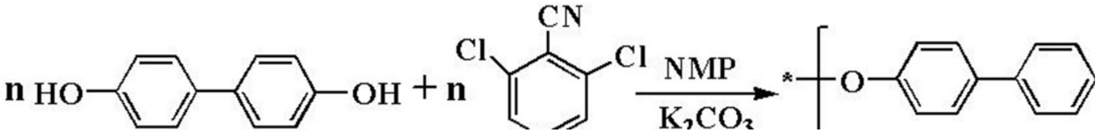

Figure S1. Synthetic route to the PEN.

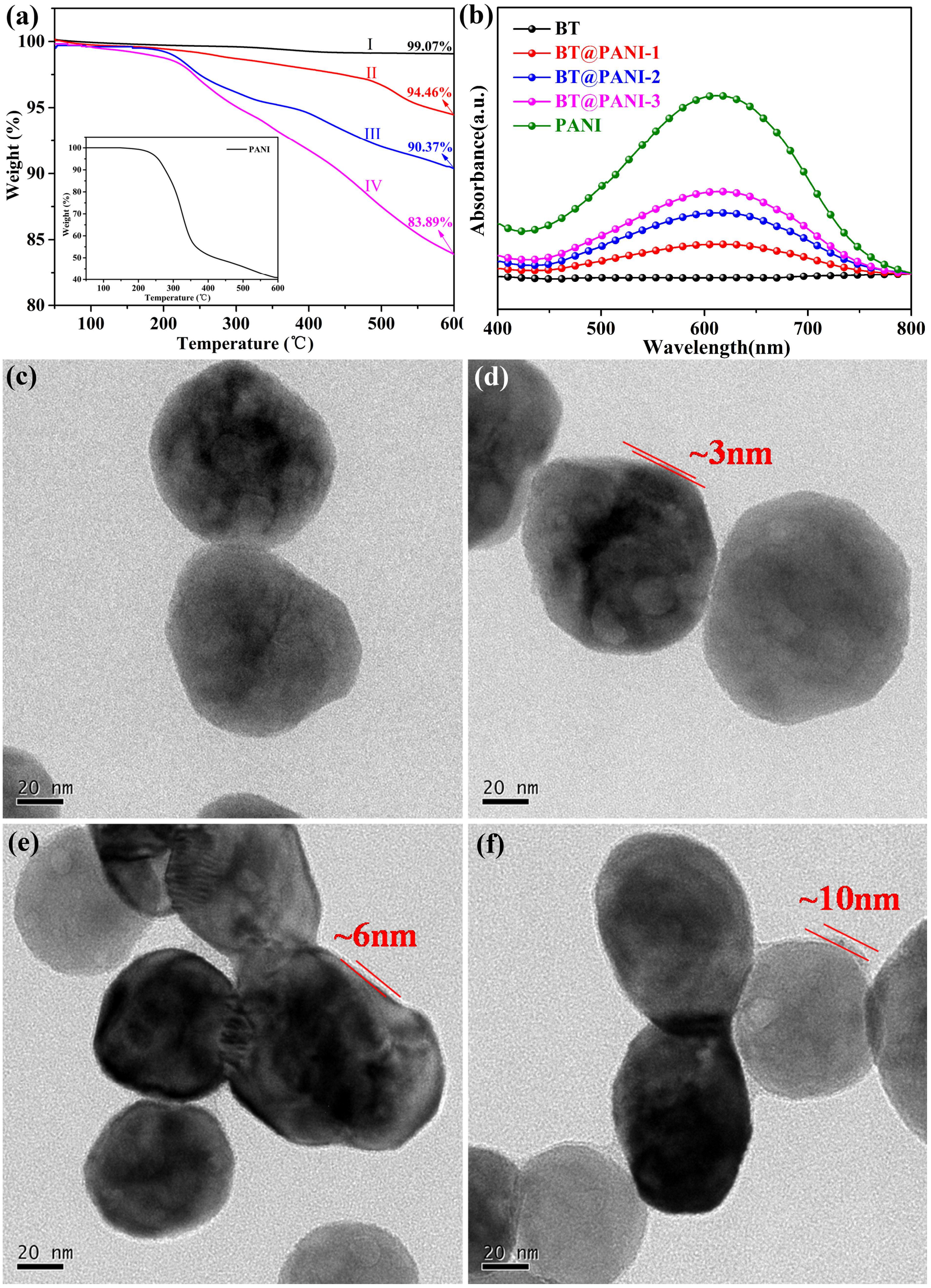

Figure S2. The characterization of nanoparticles: (a) TGA curves (I: BT, II: BT@PANI-1, III: BT@PANI-2, IV: BT@PANI-3); (b) UV-vis spectra; TEM image of (c) BT; (d) BT@PANI-1; (e) BT@PANI-2; (f) BT@PANI-3.

Table S1. The dielectric constant, dielectric loss, breakdown strength and energy density of the PEN/BT@PANI composite films at 1 kHz (20 wt%).

	PEN/BT	PEN/BT@PANI-1	PEN/BT@PANI-2	PEN/BT@PANI-3
Dielectric constant (1 kHz)	9.82	9.55	9.35	8.83
Dielectric loss (1 kHz)	0.023	0.020	0.019	0.019
Breakdown strength (kV/mm)	160.2	177.6	181.4	183.4
Energy density (J/cm^3)	1.11	1.33	1.36	1.31

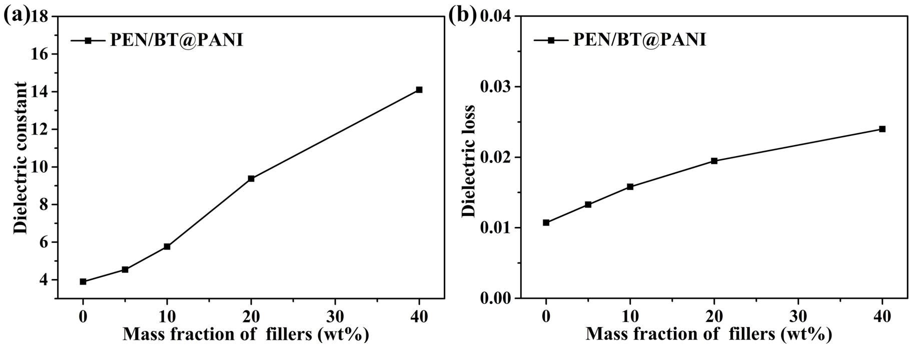

Figure S3. The dielectric constant (a) and dielectric loss (b) at 1 kHz of the PEN based nanocomposite films.
